# Mathematical modeling and quantitative analysis of HIV-1 Gag trafficking and polymerization

**DOI:** 10.1371/journal.pcbi.1005733

**Published:** 2017-09-18

**Authors:** Yuewu Liu, Xiufen Zou

**Affiliations:** 1 School of Mathematics and Statistics, Wuhan University, Computational Science Hubei Key Laboratory, Wuhan University, Wuhan, China; 2 College of Science, Hunan Agricultural University, Hunan, China; Helmholtz-Zentrum fur Infektionsforschung GmbH, GERMANY

## Abstract

Gag, as the major structural protein of HIV-1, is necessary for the assembly of the HIV-1 sphere shell. An in-depth understanding of its trafficking and polymerization is important for gaining further insights into the mechanisms of HIV-1 replication and the design of antiviral drugs. We developed a mathematical model to simulate two biophysical processes, specifically Gag monomer and dimer transport in the cytoplasm and the polymerization of monomers to form a hexamer underneath the plasma membrane. Using experimental data, an optimization approach was utilized to identify the model parameters, and the identifiability and sensitivity of these parameters were then analyzed. Using our model, we analyzed the weight of the pathways involved in the polymerization reactions and concluded that the predominant pathways for the formation of a hexamer might be the polymerization of two monomers to form a dimer, the polymerization of a dimer and a monomer to form a trimer, and the polymerization of two trimers to form a hexamer. We then deduced that the dimer and trimer intermediates might be crucial in hexamer formation. We also explored four theoretical combined methods for Gag suppression, and hypothesized that the N-terminal glycine residue of the MA domain of Gag might be a promising drug target. This work serves as a guide for future theoretical and experimental efforts aiming to understand HIV-1 Gag trafficking and polymerization, and might help accelerate the efficiency of anti-AIDS drug design.

## Author summary

The human immunodeficiency virus (HIV-1) is a retrovirus that causes acquired immunodeficiency syndrome (AIDS), an infectious disease with high annual mortality. Gag protein is the major structural protein of HIV-1 and can self-assemble into the HIV-1 sphere shell. Therefore, an in-depth understanding of Gag protein trafficking and polymerization is important for gaining further insights into the mechanisms of HIV-1 replication and the design of antiviral drugs. Through mathematical modeling, optimization and quantitative analysis, we hypothesized the budding and release time of virus-like particles and revealed that the dimer and trimer intermediates might be crucial in hexamer formation. We also concluded that the predominant pathways in hexamer formation might be the polymerization of two monomers to form a dimer, the polymerization of a dimer and a monomer to form a trimer, and the polymerization of two trimers to form a hexamer. Our analysis also suggested that the N-terminal glycine residue of the MA domain of Gag might be a promising drug target. These results serve as a guide for further theoretical and experimental efforts aiming to understand HIV-1 Gag trafficking and polymerization and could aid anti-AIDS drug design.

## Introduction

Gag protein (Gag) is the major structural polyprotein of HIV-1 and is synthesized in large amounts in the cytoplasm. Gag diffuses freely within the cytoplasm, hijacks the molecular motors, and moves along microtubules to the cytosolic side of the plasma membrane (PM) domain [1, 2]. Underneath the PM, the immature HIV-1 Gag shell assembles in a radial arrangement. Gag is composed of six constitutive components: the N-terminal matrix (MA) domain, the capsid (CA) domain, the first spacer peptide (SP1), the nucleocapsid (NC) domain, a second spacer peptide (SP2) and the p6 (p6) domain. During the phase of HIV-1 maturation, Gag disconnects from the MA and reassembles to form the cone-shaped viral core. Therefore, Gag is necessary for HIV-1 replication, interfering with the trafficking and assembly of Gag has been a focus of research [3, 4].

In the field of theoretical research, Liu et al. [5] first proposed a convection-diffusion equation model to explore the transport of Gag monomers in the cytoplasm. Based on the experimental finding of a monomer-dimer equilibrium in solution under certain biochemical conditions [6], Wang et al. [7] presented a model studying the transport of Gag monomers and trimers in the cytoplasm. These researchers analyzed the relationship between the timing of the initial appearance of HIV-1 capsid on the PM and the various model parameters. Sadre-Marandi et al. [8] and Liu et al. [9] simulated HIV-1 viral capsid assembly through dynamical systems.

A recent study on the events initiating HIV-1 Gag assembly was conducted by Kutluay et al. [10]. These researchers presented quantitative descriptions of monomers and multimers in the cytoplasm and PM, respectively, and demonstrated that only monomer and low-order multimers (e.g., dimer) of Gag were found in the cytoplasm, and that high-order multimers were formed only underneath the PM. In addition, these researchers studied two mutations of Gag: a mutated version of Gag-GFP that lacked the CTD of the CA of Gag (Gag-dCTD) and a mutation of the N-terminal glycine residue of the MA to alanine (Gag-G2A).

To the best of our knowledge, the reported models [5, 7, 11–13] focus only on Gag trafficking in the cytoplasm and do not simultaneously consider the polymerization of Gag underneath the PM. Therefore, we developed a reaction-advection-diffusion equation model to describe the trafficking of two particles and the polymerization of various particles. In our model, we focus on the following aspects:

- The transformation between monomers and dimers in the cytoplasm.
- The trafficking of monomers and dimers in the cytoplasm.
- The polymerization of monomers, dimers, trimers, tetramers, pentamers and hexamers of Gag underneath the PM.

We first estimated the parameters of the model based on experiment data [10] and assessed the robustness of the model. We subsequently applied this model to two mutation cases: Gag-dCTD and Gag-G2A. We also predicted the budding and release time of HIV-1 virus-like particles (VLPs). Using our model, we then analyzed the weight of the pathways involved in the polymerization reactions and deduced the key intermediates in hexamer formation. Moreover, we explored four theoretical combined methods for suppressing the Gag concentration and identified a promising drug target.

This work will lead to a better understanding of the dynamics of Gag-Gag interaction and Gag trafficking, which are important in the emergence of HIV, and it might provide theoretical guidance for the design of antiretroviral drugs.

## Materials and methods

### Mathematical model

**Fig 1:**
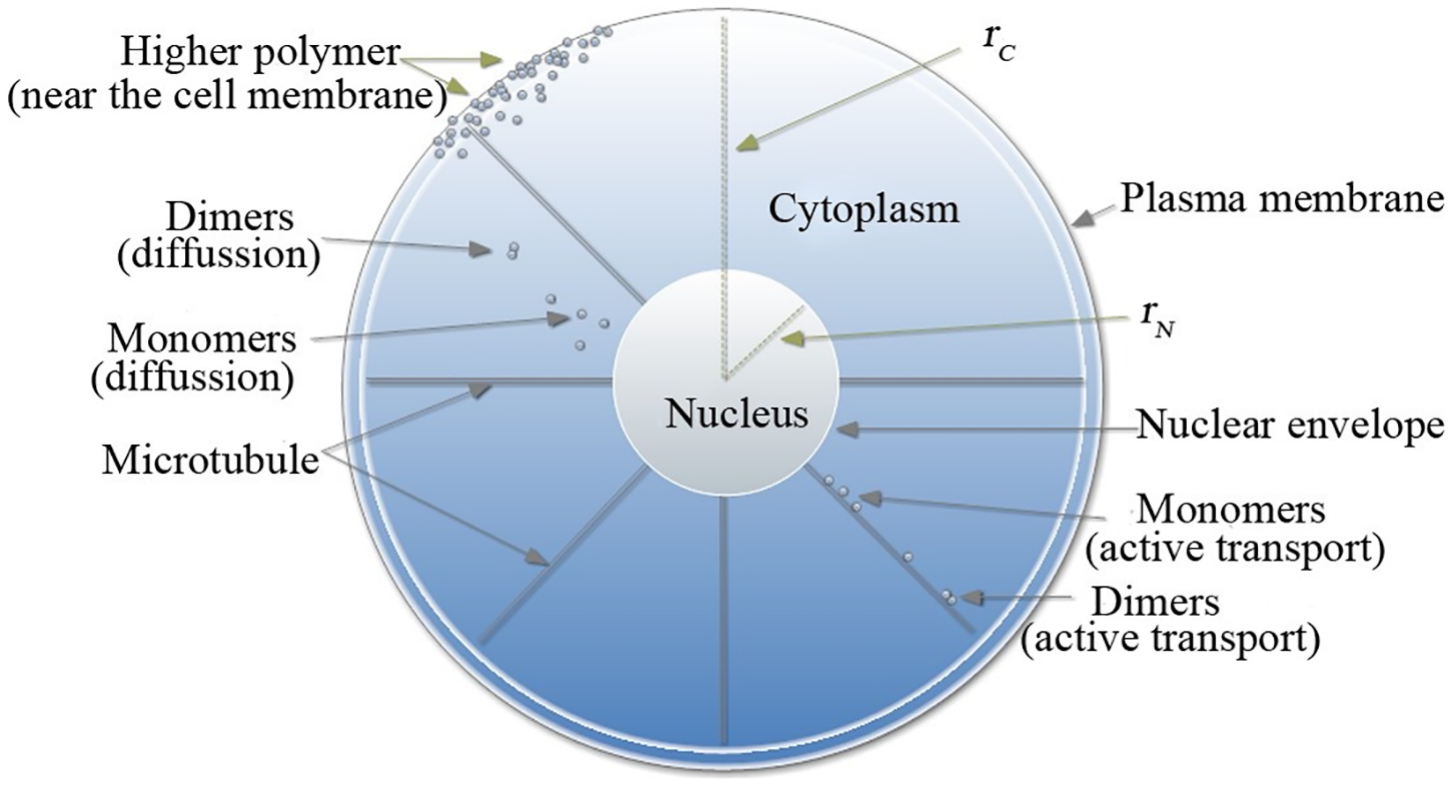
Schematic representation of a model cell. *r*_*N*_, *r*_*C*_ are the nuclear and cellular radius, respectively. In the cytoplasm (*r*_*N*_ < *r* < *r*_*C*_), Gag monomers can aggregate to dimers, but not higher-order polymers. Monomers and dimers are transported actively by molecular motors along microtubules and can diffuse along the direction of the concentration gradient. Underneath the plasma membrane (*r*_*C*_), monomers can be polymerized to hexamers.

This work aimed to assess the Gag trafficking in the cytoplasm and Gag polymerization underneath the PM. The schematic diagram used to develop the mathematical model is shown in Fig 1. Several assumptions were made to simplify the model:

**A1** The cytoplasm is an annulus [5, 14].

**A2** In the cytoplasm, monomers can aggregate into dimers, but can not form higher-order polymers [10, 15–18].

**A3** Monomers and dimers are transported to the PM along microtubules by molecular motors (e.g., kinesin and dynein), and can also diffuse freely in the cytoplasm [5, 11, 13, 14].

**A4** Gag is synthesized by ribosomes attached to the endoplasmic reticulum (ER). A large amount of ER is located in the perinuclear region, and a slight amount of ER is found underneath the PM. We approximated that the density of ER decreased exponentially from the perinuclear region to the PM. In addition, newly synthesized Gag monomers are distributed throughout the cytoplasm, but concentrated in the perinuclear region [19]. Based on these assumptions, we assumed that the synthesis rate of Gag decreased (roughly) exponentially from the perinuclear region to the PM.

**A5** Gag is observed on the PM within 5-10 minutes post-synthesis [20, 21]. Typically, a period of 5-6 minutes is required to complete the assembly of a single VLP [22]. The budding and release time of a VLP is approximately 6 hours [22]. Furthermore, some researchers [23, 24] have concluded that the hexamer is the building block of HIV-1. Therefore, we hypothesized that Gag monomers can only aggregate into dimers, trimers, tetramers, pentamers, and hexamers during the first 30 minuntes, and this assumption was mainly used to build the mathematical model (Eq (6)) in the boundary (PM).

**A6** Gag polymers degrade with different degradation rates [21].

**A7** Some molecular motors (e.g., kinesin) move unidirectionally from the microtubule-organizing center to the cell periphery, whereas others (e.g., dynein) move toward the cell nucleus [25, 26]. During the process of egress, the difference between the outward and inward speeds is denoted the velocity of egress [5, 14].

In the cytoplasm (*r*_*N*_ < *r* < *r*_*C*_), the chemical reactions involving monomers and dimers can be described as follows:

$$2Gag\overset{k_{1}}{\underset{k_{1}^{\prime }}{⇌}}Gag_{2},\phi \overset{g_{1}\left(r\right)}{\to }Gag,Gag\overset{d_{1}}{\to }\phi ,Gag_{2}\overset{d_{2}}{\to }\phi$$ (1)

where *Gag* is a monomer and *Gag*_2_ is a dimer. Definitions of the variables and symbols are provided in Table 1.

**Table 1:** Nomenclature of the mathematical symbols.

Symbol	Description
*P*_1_(*r*, *t*)	The monomer concentration at the radial position *r* and time *t*
*P*_2_(*r*, *t*)	The dimer concentration at the radial position *r* and time *t*
*T*	The elapsed time of the experiment
*k*_1_	The on-rate constant of monomer
$k_{1}^{\prime }$	The off-rate constant of dimer
*g*_1_(*r*)	The generation rate of monomer at the radial position *r*
*D*_1_	The diffusion coefficient of monomer
*D*_2_	The diffusion coefficient of dimer
*s*_1_	The average transfer speed of monomer
*s*_2_	The average transfer speed of dimer
*d*_1_	The degradation rate of monomer
*d*_2_	The degradation rate of dimer

In rectangular coordinates, the transport velocity of monomer is *v*_1_ = (*v*_1*x*_, *v*_1*y*_), where *v*_1*x*_ and *v*_1*y*_ are the velocities along the *x* and *y* directions, respectively. For simplicity, we switched the problem to polar coordinates. Thus, the velocity of monomer along the radial direction is denoted by *s*_1_, and the angle between *s*_1_ and the polar axis is denoted by *θ*_1_. Therefore, *v*_1_ = (*s*_1_ cos *θ*_1_, *s*_1_ sin *θ*_1_).

The total flux of monomer transportation includes both convective and diffusive transport: ∇ ⋅ (*v*_1_*P*_1_ − *D*_1_∇*P*_1_). In polar coordinates, the above equation yields:

$$\begin{array}{c} \nabla \cdot (v_{1}P_{1}-D_{1}\nabla P_{1})=\frac{1}{r}\frac{\partial }{\partial r}(s_{1}rP_{1}-D_{1}r\frac{\partial P_{1}}{\partial r}) \end{array}$$

The equation for total dimer flux has the same form. Based on the mass conservation law and mass action law [27], we obtain the following reaction-diffusion-transport equations:

$$\{\begin{array}{ll} \frac{\partial P_{1}}{\partial t}= & \frac{1}{r}\frac{\partial }{\partial r}\left(D_{1}r\frac{\partial P_{1}}{\partial r}-s_{1}rP_{1}\right)+2k_{1}^{\prime }P_{2}-2k_{1}P_{1}^{2}+g_{1}\left(r\right)-d_{1}P_{1}\\ \frac{\partial P_{2}}{\partial t}\;= & \frac{1}{r}\frac{\partial }{\partial r}\left(D_{2}r\frac{\partial P_{2}}{\partial r}-s_{2}rP_{2}\right)+k_{1}P_{1}^{2}-k_{1}^{\prime }P_{2}-d_{2}P_{2} \end{array}$$ (2)

where *t* ∈ (0, *T*), *r* ∈ (*r*_*N*_, *r*_*C*_).

### Boundary and initial conditions

At the outer membranes of the nucleus (*r* = *r*_*N*_), impermeable wall boundary conditions are considered as follows:

$$\begin{array}{c} \left\{ \begin{array}{cc} & D_{1}r\frac{\partial P_{1}}{\partial r}-s_{1}rP_{1}=0\\ & D_{2}r\frac{\partial P_{2}}{\partial r}-s_{2}rP_{2}=0 \end{array},\;r=r_{N}\right. \end{array}$$ (3)

Gag proteins gathered at the “Gag hotspots” underneath the PM, which has a thickness of approximately 20 nm [14]. This domain of “Gag hotspots” is considered a volume, and we set it as the boundary of our model, similarly to the strategy used in a previous study [28]. Therefore, the concentrations of polymers on the boundary reflect all the volume concentrations, which have the same units at *P*_1_ and *P*_2_ in the cytoplasm.

At the PM (*r* = *r*_*C*_), monomer transport includes both convection and diffusion, and the same is true for dimer transport. However, underneath the PM, a myristoyl group of Gag can attach to the PM, resulting a weaker free diffusion of Gag compared with that in the cytoplasm. Therefore, we reduced the diffusion coefficient in the cytoplasm by *k*_*D*_ to obtain the diffusion coefficient underneath the PM. Gag proteins are transported by molecular motors along microtubules, which are found throughout the cytoplasm, but are relatively rare underneath the PM. Therefore, the velocity of Gag loading to the PM is relatively small. We thus also reduced the velocity in the cytoplasm by *k*_*s*_ to obtain the transport coefficient underneath the PM. Therefore, the monomer and dimer fluxes can be computed as follows:

$$\begin{array}{c} \begin{array}{cc} & k_{D}D_{1}r\frac{\partial P_{1}}{\partial r}-k_{s}s_{1}rP_{1}\;\;\text{and}\;\;k_{D}D_{2}r\frac{\partial P_{2}}{\partial r}-k_{s}s_{2}rP_{2} \end{array},\;r=r_{C} \end{array}$$ (4)

When the termination time *T* is approximately 30 minutes, Gag monomers can only aggregate into dimers, trimers, tetramers, pentamers, and hexamers based on assumption A5. Thus, the interactions among monomers, dimers, trimers, tetramers, pentamers, and hexamers underneath the PM (*r* = *r*_*C*_) were studied, and all possible chemical reactions based on the step-growth polymerization [29] are the following:

$$\begin{array}{l} Gag_{i}+Gag_{j}\overset{k_{ij}}{\underset{k_{ij}^{\prime }}{⇌}}Gag_{i+j},i\leq j,i+j\leq 6,i,j=1,2,\cdots ,6,\\ Gag_{n}\overset{d_{n}}{\to }\phi ,n=1,2,\cdots ,6, \end{array}$$ (5)

where *Gag*_*i*_ is a polymer with *i* monomers, *k*_*ij*_ is the on-rate constant, $k_{ij}^{\prime }$ is the off-rate constant and *d*_*n*_ is the degradation rate of *n*-mers.

By combining with the above mentioned chemical reactions and Eq (4), the following boundary conditions at the PM are obtained:

$$\begin{array}{c} \;\{\begin{array}{cc} & \frac{\partial P_{1}}{\partial t}=\frac{1}{r}\frac{\partial }{\partial r}(k_{D}D_{1}r\frac{\partial P_{1}}{\partial r}-k_{s}s_{1}rP_{1})\text{+}2{k^{\prime }}_{11}P_{2}-2k_{11}P_{1}^{2}+{k^{\prime }}_{12}P_{3}-k_{12}P_{1}P_{2}+\\ & \;\;\;\;\;\;\;\;{k^{\prime }}_{13}P_{4}-k_{13}P_{1}P_{3}+{k^{\prime }}_{14}P_{5}-k_{14}P_{1}P_{4}+{k^{\prime }}_{15}P_{6}-k_{15}P_{1}P_{5}-d_{1}P_{1}\\ & \frac{\partial P_{2}}{\partial t}=\frac{1}{r}\frac{\partial }{\partial r}(k_{D}D_{2}r\frac{\partial P_{2}}{\partial r}-k_{s}s_{2}rP_{2})\text{+}k_{11}P_{1}^{2}-{k^{\prime }}_{11}P_{2}+{k^{\prime }}_{12}P_{3}-k_{12}P_{1}P_{2}+\\ & \;\;\;\;\;\;\;\;2{k^{\prime }}_{22}P_{4}-2k_{22}P_{2}^{2}+{k^{\prime }}_{23}P_{5}-k_{23}P_{2}P_{3}+{k^{\prime }}_{24}P_{6}-k_{24}P_{2}P_{4}-d_{2}P_{2}\\ & \frac{\partial P_{3}}{\partial t}=k_{12}P_{1}P_{2}-{k^{\prime }}_{12}P_{3}+{k^{\prime }}_{13}P_{4}-k_{13}P_{1}P_{3}+{k^{\prime }}_{23}P_{5}-k_{23}P_{2}P_{3}+2{k^{\prime }}_{33}P_{6}-\\ & \;\;\;\;\;\;\;\;2k_{33}P_{3}^{2}-d_{3}P_{3}\\ & \frac{\partial P_{4}}{\partial t}=k_{22}P_{2}^{2}-{k^{\prime }}_{22}P_{4}+k_{13}P_{1}P_{3}-{k^{\prime }}_{13}P_{4}+{k^{\prime }}_{14}P_{5}-k_{14}P_{1}P_{4}+{k^{\prime }}_{24}P_{6}-\\ & \;\;\;\;\;\;\;\;k_{24}P_{2}P_{4}-d_{4}P_{4}\\ & \frac{\partial P_{5}}{\partial t}=k_{23}P_{2}P_{3}-{k^{\prime }}_{23}P_{5}+k_{14}P_{1}P_{4}-{k^{\prime }}_{14}P_{5}+{k^{\prime }}_{15}P_{6}-k_{15}P_{1}P_{5}-d_{5}P_{5}\\ & \frac{\partial P_{6}}{\partial t}=k_{33}P_{3}^{2}-{k^{\prime }}_{33}P_{6}+k_{24}P_{2}P_{4}-{k^{\prime }}_{24}P_{6}+k_{15}P_{1}P_{5}-{k^{\prime }}_{15}P_{6}-d_{6}P_{6} \end{array} \end{array}$$ (6)

The initial conditions are *P*_*i*_ = 0, *i* = 1, 2, ⋯, 6, which are based on the experimental data [10].

### A reduced model

The nine polymerization reactions (5) underneath the PM and one polymerization reaction (1) in the cytoplasm have 20 parameters, including *k*_*i*,*j*_, $k_{i,j}^{\prime }$, *i* ≤ *j*, *i*+*j* ≤ 6, *i*, *j* = 1, 2, ⋯, 6. To decrease the number of these parameters, we adopted the strategy described by Zlotnick et al. [30–32] in their study of the assembly kinetics of virus capsids.

Zlotnick et al. used a system of equations to simulate the sequential aggregation of free building blocks into virus capsids. To reduce the number of parameters, these researchers developed a formula [30, 31] that mapped the on-rate constant to the off-rate constant. In our study, Gag proteins are aggregated to form a hexamer, and this process has a lot in common with virus capsid assembly. For example, the virus capsid and the subunit in the work conducted by Zlotnick et al. correspond to the hexamer and the low-order polymer serving as one of the two reactants in each polymerization reaction in our work, respectively.

For the polymerization reactions (5), the association constant *K*_*i*+*j*_ of *Gag*_*i*+*j*_ can be separated into two statistical components *SI*_*i*,*j*_ and *S*_*i*,*j*_, and a non-statistical association constant $K_{i+j}^{\prime }$. These are related by the following function:

$$\begin{array}{c} K_{i+j}=\frac{k_{i,j}}{k_{i,j}^{\prime }}=SI_{i,j}S_{i,j}K_{i+j}^{\prime } \end{array}$$ (7)

where the statistical factor *SI*_*i*,*j*_ describes the degeneracy of the incoming subunit. The second statistical factor *S*_*i*,*j*_ can be treated as the ratio of two factors: the number of pathways for the formation of *Gag*_*i*+*j*_ from *Gag*_*i*_ and *Gag*_*j*_ and the number of pathways for the dissociation of *Gag*_*i*+*j*_ to *Gag*_*i*_ and *Gag*_*j*_. $K_{i+j}^{\prime }$ is a function of the number of contacts formed, i.e.,

$$\begin{array}{c} {K^{\prime }}_{i+j}=e^{-c_{i,j}\Delta G/RT} \end{array}$$ (8)

where *c*_*i*,*j*_ is the number of contacts of *Gag*_*i*_ and *Gag*_*j*_, Δ*G* is the free energy associated with the formation of a contact, -2.72 *kcal* *mol*^−1^, *R* is the gas constant, 1.987 *cal* deg^−1^*mol*^−1^, and *T* is the temperature in Kelvin, 298*K*.

As determined by substituting Eq (8) into Eq (7), *k*′_*i*,*j*_ can be given by the parameter *k*_*i*,*j*_ based on the following function:

$$\begin{array}{c} {k^{\prime }}_{i,j}=\frac{k_{i,j}}{{SI}_{i,j}S_{i,j}e^{-c_{i,j}\Delta G/RT}} \end{array}$$ (9)

**Fig 2:**
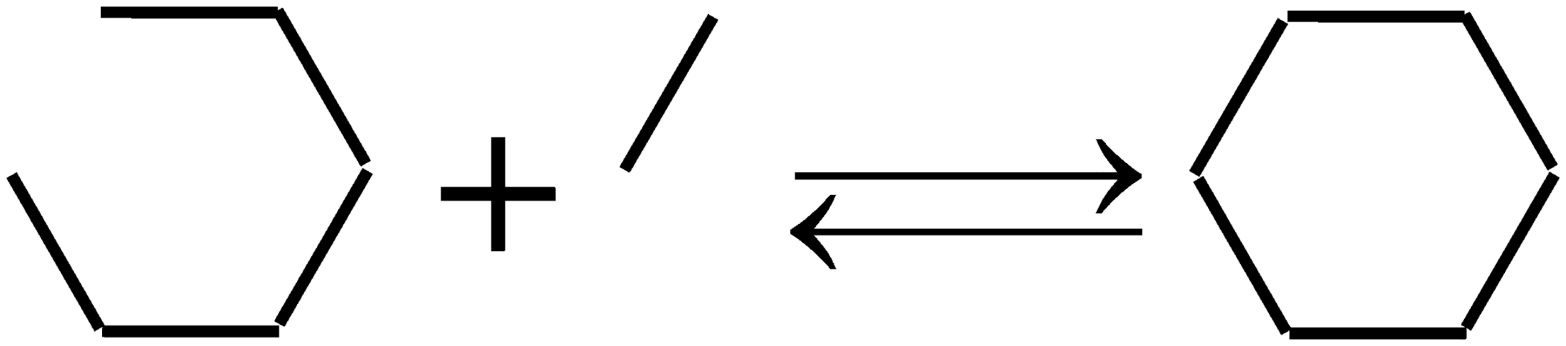
A cartoon to show the polymerization reaction of a monomer and a pentamer.

As an example, the evaluation of $k_{1,5}^{\prime }$ is described. Owen et al. [23] found that Gag monomers could create a hexameric ring, which was believed to serve as the building block of HIV-1, thus the hexamer can be considered a hexagon from the perspective geometry. Then, *i* edges next to each other are removed from the hexagon, and we used these as the geometry of the *i*-mers of Gag. Fig 2 shows the reaction in which a monomer and a pentamer aggregate to form a hexamer. There is only one way to add a monomer to a pentamer to form a hexamer, and there are six ways to dissociate a hexamer to a monomer and a pentamer, thus *S*_1,5_ = 1/6. The incoming subunit is the monomer, thus *SI*_1,5_ = 1. Two contacts (*c*_*i*,*j*_ = 2) are made in forming of *Gag*_6_, resulting in a free energy of 2Δ*G*. Thus,

$$\begin{array}{c} {k^{\prime }}_{1,5}=\frac{k_{1,5}}{1\times 1/6\times e^{-2\Delta G/RT}}. \end{array}$$

For the polymerization reactions (5), *SI*_*i*,*j*_, *S*_*i*,*j*_ and *c*_*i*,*j*_ are counted and these are listed in Table 2.

**Table 2:** Polymerization reactions and statistical factors.

Polymerization reaction	Number of association ways ^1^	Number of dissociation ways	*S*_*i*,*j*_	*c*_*i*,*j*_	*S*_*in*_
Gag + Gag ↔ Gag_2_	2/2 ^2^	1	1	1	1
Gag + Gag_2_ ↔ Gag_3_	2	2	1	1	1
Gag + Gag_3_ ↔ Gag_4_	2	2	1	1	1
Gag + Gag_4_ ↔ Gag_5_	2	2	1	1	1
Gag + Gag_5_ ↔ Gag_6_	1	6	1/6	2	1
Gag_2_ + Gag_2_ ↔ Gag_4_	2/2	1	1	1	2
Gag_2_ + Gag_3_ ↔ Gag_5_	2	2	1	1	2
Gag_2_ + Gag_4_ ↔ Gag_6_	1	6	1/6	2	2
Gag_3_ + Gag_3_ ↔ Gag_6_	1	3	1/3	2	3

^1^ A computed example is shown in the above Section.

^2^ The factor of 1/2 is the symmetry factor because two reactants are the same.

We only need to optimize the values of *k*_*i*,*j*_, because $k_{i,j}^{\prime }$ can be obtained by the above-mentioned function (9). Therefore, the 20 parameters for nine polymerization reactions (5) underneath the PM and the single polymerization reaction (1) in the cytoplasm are cut by half. Combined with two proportionality coefficients *k*_*D*_, *k*_*s*_ and the velocity *s*1 of Gag-G2A, this results in 13 parameters that needed to be optimized. Thus, the number of parameters to be optimized was substantially decreased.

### Model parameters

The radius of the cell nucleus is ∼5 *μm* [5, 14], and the radius of the cell is ∼10 *μm* [5, 14].

The diffusion coefficient for an “average” (3 − 6 *nm* diameter) protein in the cytoplasm is 5 − 15 *μm*^2^/*s* [33]. The Gag monomer is a highly extended rod with a length of ∼20 *nm* and a width of 2 − 3 *nm* [5], resulting in an average mean of 11.25 *nm* between the length and width. According to the Stokes-Einstein equation, the diffusion coefficient is inversely proportional to the diameter. Therefore, we estimated the diffusion coefficient of Gag as ∼4 *μm*^2^/*s*. Similarly, the diffusion coefficient for a Gag dimer is approximately half of the corresponding value for a Gag monomer.

The velocities of the active transport of a monomer and a dimer are approximately equal to the velocity of the molecular motor (∼1 *μm*/*s* [33]) in the cytoplasm.

Tritel et al. [34] found that 80% of Gag disappeared within 2 hours after synthesis. Therefore, we estimated that the degradation rate of a monomer was ~ 2.236 × 10^−4^/*s*, and the degradation rate of *i*-mers was thus ∼2.236 × 10^−4^/*i* /*s*.

The above-described parameter values estimated by the experimentally measured data are listed in Table 3.

**Table 3:** Some parameter values estimated by the experimentally measured data.

Parameter	Unit	Value	Reference
*r*_*N*_	*μm*	5	Liu et al. [5], Munoz-Alicea et al. [14]
*r*_*C*_	*μm*	10	Liu et al. [5], Munoz-Alicea et al. [14]
*D*_1_	*μm*^2^/*s*	4	Liu et al. [5], Moran et al. [33]
*D*_2_	*μm*^2^/*s*	2	Liu et al. [5], Moran et al. [33]
*s*1	*μm*/*s*	1	Moran et al. [33]
*s*2	*μm*/*s*	1	Moran et al. [33]
*d*1	/*s*	2.236 × 10^−4^	Tritel et al. [34]
*d*2	/*s*	1.118 × 10^−4^	Tritel et al. [34]
*d*3	/*s*	0.745 × 10^−4^	Tritel et al. [34]
*d*4	/*s*	0.559 × 10^−4^	Tritel et al. [34]
*d*5	/*s*	0.447 × 10^−4^	Tritel et al. [34]
*d*6	/*s*	0.373 × 10^−4^	Tritel et al. [34]

### Numerical methods for solving mathematical model

We adopted the Crank-Nicolson method for discretizing the convection-diffusion-reaction equations to form nonlinear equations, and then used Newton’s method to solve them.

First, we discretized the system of convection-diffusion-reaction equations using the Crank-Nicolson method. Let the time step and grid size of the radius be Δ*t* and Δ*r*, respectively. Then, the *i* − *mers* concentration is denoted by $P_{i,n}^{k}=P_{i}\left(r_{N}+n\Delta r,k\Delta t\right)$. The derivatives of $P_{i,n}^{k}$ with respect to *t* and *r* are discretized as follows:

$$\begin{array}{c} \;\begin{array}{cc} & \frac{\partial P_{i}}{\partial t}=\frac{P_{i,n}^{k+1}-P_{i,n}^{k}}{\Delta t}\\ & \frac{\partial P_{i}}{\partial r}=\frac{1}{2}(\frac{P_{i,n+1}^{k+1}-P_{i,n-1}^{k+1}}{2\Delta r}+\frac{P_{i,n+1}^{k}-P_{i,n-1}^{k}}{2\Delta r})\\ & \frac{\partial^{2}P_{i}}{\partial r^{2}}=\frac{1}{2}(\frac{P_{i,n+1}^{k+1}-2P_{i,n}^{k+1}+P_{i,n-1}^{k+1}}{{\left(\Delta r\right)}^{2}}+\frac{P_{i,n+1}^{k}-2P_{i,n}^{k}+P_{i,n-1}^{k}}{{\left(\Delta r\right)}^{2}}) \end{array} \end{array}$$ (10)

The system of convection-diffusion-reaction equations (Eqs 2, 3 and 6) were discretized according to the above-mentioned rules (10), and the resulting equation can be rewritten using vectors as

$$\begin{array}{c} A\left(\Delta t,\Delta r,r\right)X^{k+1}+F\left(r,X^{k+1}\right)=B\left(\Delta t,\Delta r,r\right)X^{k} \end{array}$$ (11)

where *N* is the grid number of the radius, $X^{k}={\left(P_{1,0}^{k},P_{1,1}^{k},\cdots ,P_{1,N}^{k},P_{2,0}^{k},P_{2,1}^{k},\cdots ,P_{2,N}^{k},\cdots ,\;P_{6,0}^{k},P_{6,1}^{k},\cdots ,P_{6,N}^{k}\right)}^{T}$, *A* and *B* are all (6*N* + 6) × (6*N* + 6) matrices that depend on the time step Δ*t*, grid size Δ*r* and radius *r*. *F* is the nonlinear part, which depends on *r* and *X*^*k*+1^.

At each step, Eq (11) is a nonlinear algebraic equation that can be solved using Newton’s method. The following process is repeated

$$\begin{array}{c} \begin{array}{cc} & F_{1}\left(\Delta t,\Delta r,r,X^{k},X^{k-1}\right)=A\left(\Delta t,\Delta r,r\right)X^{k}+F\left(r,X^{k}\right)-B\left(\Delta t,\Delta r,r\right)X^{k-1}\\ & \Delta X^{k}={\left(\nabla F_{1}\left(\Delta t,\Delta r,r,X^{k},X^{k-1}\right)\right)}^{-1}(F_{1}\left(\Delta t,\Delta r,r,X^{k},X^{k-1}\right))\\ & X^{k+1}=X^{k}-\Delta X^{k} \end{array} \end{array}$$ (12)

until a sufficiently accurate value is reached.

The numerical algorithms were implemented in MATLAB 2009b on a personal computer. To ensure numerical accuracy, a small time step Δ*t* = 1 *s* and grid size Δ*r* = 0.025 *μm* were used. The numerical solutions converged for Δ*t* in the range from 0.5 to 36 *s* and Δ*r* in the range from 0.0063 to 0.05 *μm*, respectively.

### Identification of model parameters

**Fig 3:**
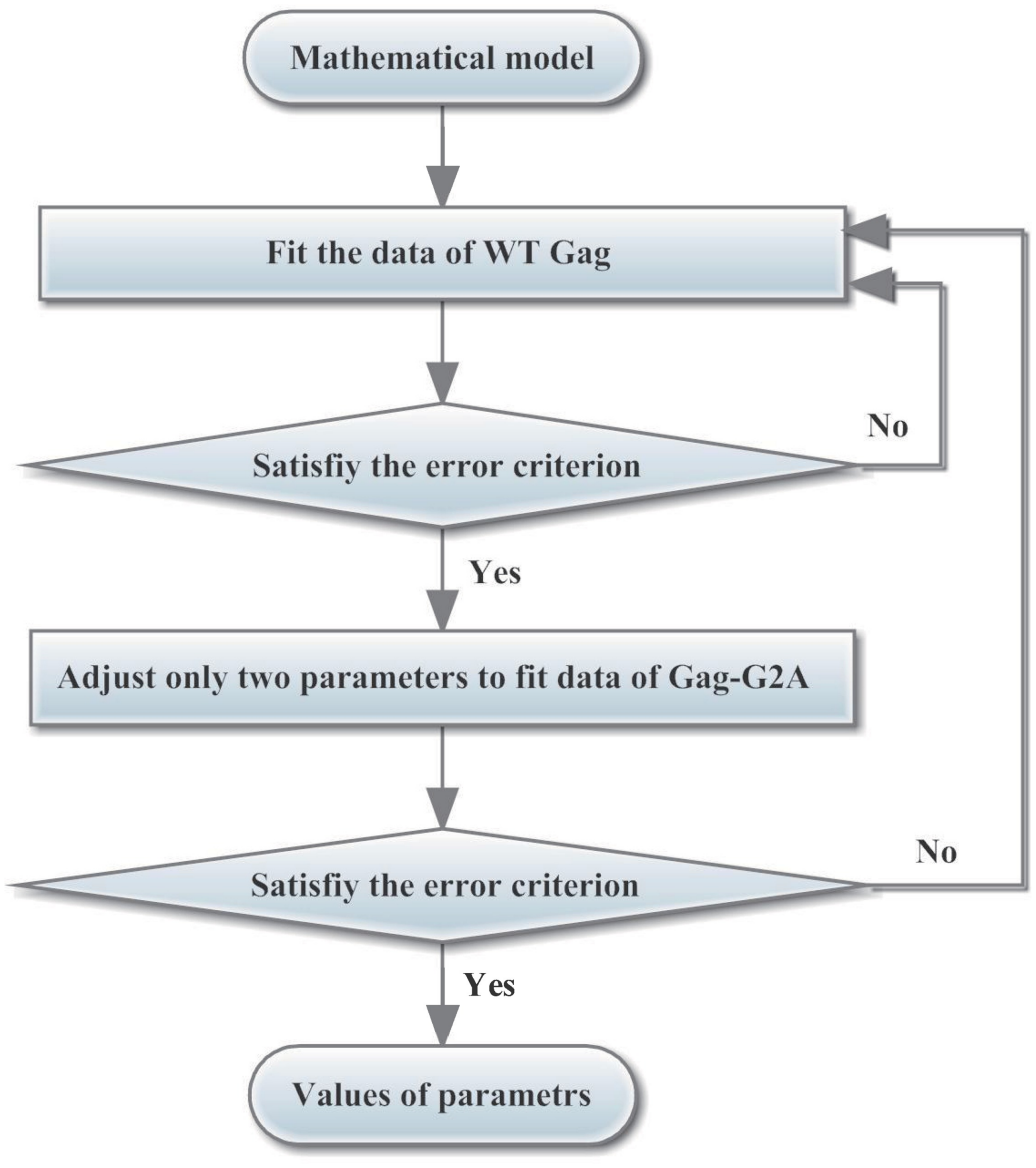
The flow chart of the parameter optimization.

Some parameters were determined from a variety of sources, as illustrated in Table 3, and the others needed to be obtained using an optimization method. For WT Gag, 12 free parameters needed to be optimized. In contrast, for Gag-G2A, only one parameter *s* needed to be optimized, and the other parameter values are equal to the corresponding values for WT Gag. Therefore, 13 parameters needed to be optimized by fitting to 16 experiment data points (eight data points for WT Gag and eight data points for Gag-G2A [10]). The flow chart of this process is shown in Fig 3. The advantage of the sequential scheme in Fig 3 is that the second object function will not be run until the first one meets the error criterion, and this process can reduce the program running time on a personal computer. If this program runs in a supercomputer with thousands of computers, other schemes for parallel computing, such as the weighted multi-objective scheme [35, 36], would be more efficient.

Thirteen parameters need to be optimized using 16 experiment data points (eight data points for WT Gag and eight data points for Gag-G2A [10]): this is the inverse problem. In addition, the measured data is always inevitable mixed with noise. In numerical computation, this problem is often ill-posed. To decrease over-parametrization and guarantee numerical stability of this optimization problem, a regularization term using the Tikhonov regularization method is generally added (Eq 13) [37–39].

The idea of regularization is to add preference to a particular solution with desirable properties [38, 40–42]. In many cases, the solution is given preference with smaller norms, and this process is known as *L*_2_ regulation. This regulation improves the conditioning of the problem, enabling a direct numerical solution. The form of regularization is given as:

$$\underset{\theta_{1}\leq \theta \leq \theta_{2}}{\text{min}}J\left(\theta \right)=\;{\left\|Y\left(\theta \right)-Y^{\left(\text{exp}\right)}\right\|}_{2}\;+\;\lambda {\left\|\theta \right\|}_{2}$$ (13)

where *θ* is the parameter vector, *θ*_1_ and *θ*_2_ are the lower and upper bounds of *θ*, respectively. *Y*(*θ*) and *Y*^(exp)^ are the calculation and the experimental data, respectively. ‖ ⋅ ‖_2_ is the Euclidean norms. λ‖*θ*‖_2_ is the regularization term, and λ is the weight coefficient, which is generally small and is set to 0.001.

Because the model and the boundary conditions are nonlinear, intelligent optimization algorithms, such as Differential Evolution (DE) and Particle Swarm Optimization(PSO), are commonly used to obtain the parameter values. Here, we use the diversity-maintained differential evolution based on a gradient local search (DMGBDE) method proposed by Xie et al. [43], which might have improved local search ability. The DMGBDE procedure can be described as follows.

1. Randomly initialize the population with N individuals.
2. Compute the objective value of each individual in the population.
3. Loop
4. Generate new individuals using the diversity-maintained mutation, select the best individual.
5. If the best individual has been renewed, perform the quasi-Newton local search around the best individual and renew it further.
6. Otherwise, perform the quasi-Newton local search around other competitive individuals and renew them further.
7. Renew the objective values.
8. If the terminating condition is reached, exit loop.
9. End loop

## Results

### Comparisons between the simulation results and experiment data

Kutluay et al. [10] used a chemical crosslinking approach to analyze the initiating events in HIV-1 assembly and genome packaging. In their experiment, 293T cells coexpressing WT Gag and HIV-1 RNA were crosslinked by treatment with EGS, a membrane-permeable crosslinker. After 30 minutes of incubation at room temperature, crosslinking was prevented by the addition of Tris-Cl. The cells were then analyzed through membrane flotation assays. Proteins from the PM and cytoplasmic fractions, including monomers, dimers, trimers, tetramers, pentamers, and hexamers, were precipitated, and their relative concentrations were obtained by western blotting.

**Fig 4:**
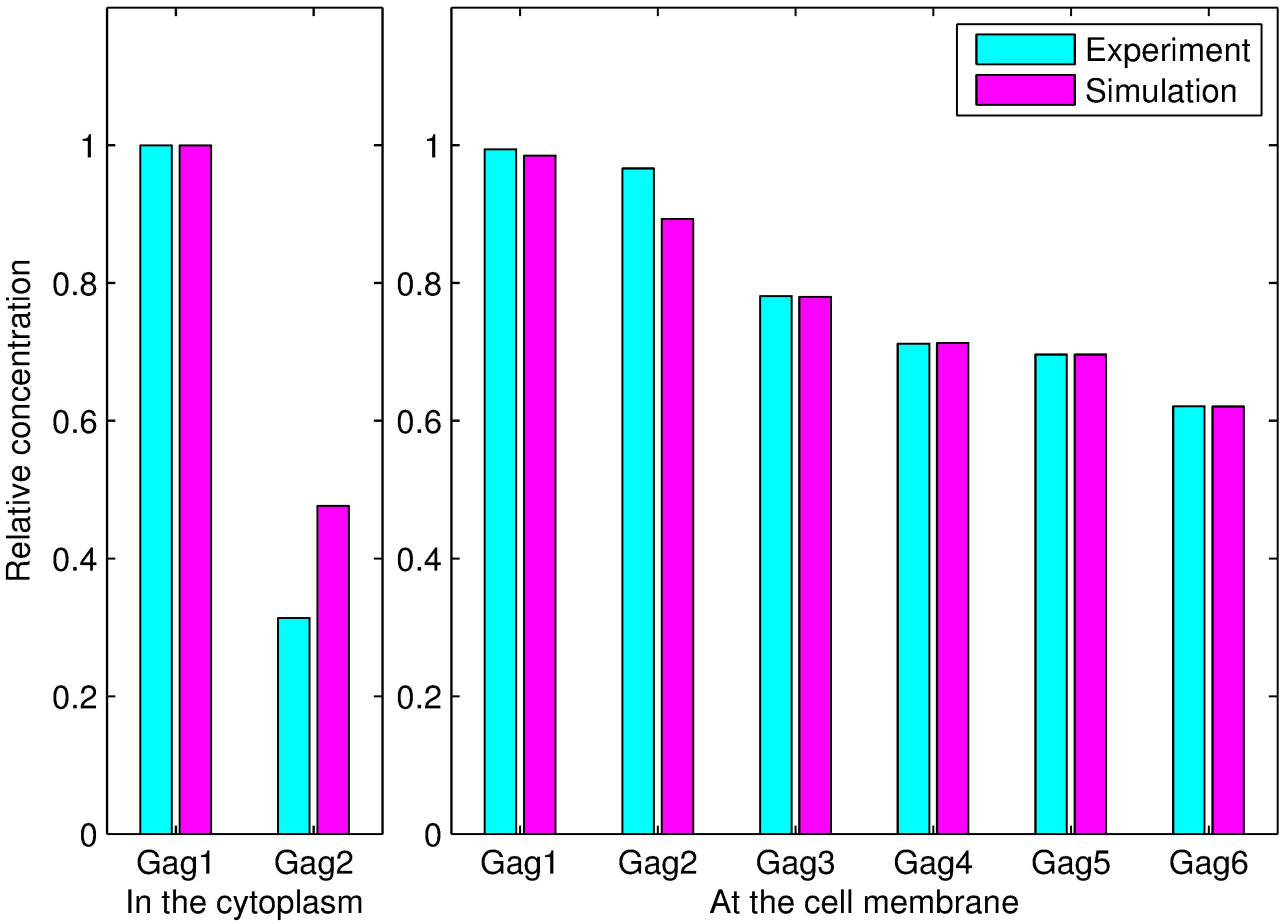
Comparisons between the simulation results and experimental data. *Gagi*(*i* = 1, 2, ⋯, 6) denotes a polymer with *i* monomers. The subfigure on the left illustrates the comparisons between the simulation and experimental results for Gag monomers and dimers in the cytoplasm. The subfigure on the right shows the differences between the simulation and experimental results for the six types of polymers at the plasma membrane. The concentration of each polymer was normalized by dividing by the concentration of Gag monomer in the cytoplasm.

The values of the parameters were optimized and are shown in Table 4. The simulated absolute concentrations and experimentally measured relative concentrations of the polymers were normalized by dividing by the concentration of Gag monomer in the cytoplasm. As shown in Fig 4, the simulation results are consistent with the experimental data.

**Table 4:** Some parameter values for WT Gag optimized by the Tikhonov regularization method with data from [10].

Parameter	Unit	Value	Range	Confidence interval ^1^
*k*_1_ ^2^	*μm*^3^/(*ymol* ⋅ *s*)	7.22 × 10^−3^	> 0	[5.28 × 10^−3^, 7.78 × 10^−3^]
*k*_11_	*μm*^3^/(*ymol* ⋅ *s*)	1.36 × 10^−4^	> 0	[1.28 × 10^−4^, 1.44 × 10^−4^]
*k*_12_	*μm*^3^/(*ymol* ⋅ *s*)	4.75 × 10^−4^	> 0	[4.33 × 10^−4^, 5.19 × 10^−4^]
*k*_22_	*μm*^3^/(*ymol* ⋅ *s*)	8.33 × 10^−5^	> 0	[7.22 × 10^−5^, 9.17 × 10^−5^]
*k*_13_	*μm*^3^/(*ymol* ⋅ *s*)	1.14 × 10^−4^	> 0	[7.78 × 10^−5^, 1.56 × 10^−4^]
*k*_23_	*μm*^3^/(*ymol* ⋅ *s*)	2.33 × 10^−4^	> 0	[2.08 × 10^−4^, 2.92 × 10^−4^]
*k*_14_	*μm*^3^/(*ymol* ⋅ *s*)	8.33 × 10^−6^ ^3^	> 0	[0, 4.72 × 10^−5^]
*k*_33_	*μm*^3^/(*ymol* ⋅ *s*)	1.22 × 10^−4^	> 0	[6.39 × 10^−5^, 1.83 × 10^−4^]
*k*_24_	*μm*^3^/(*ymol* ⋅ *s*)	1.11 × 10^−4^	> 0	[5.28 × 10^−5^, 1.22 × 10^−4^]
*k*_15_	*μm*^3^/(*ymol* ⋅ *s*)	1.64 × 10^−4^	> 0	[2.78 × 10^−5^, 3.19 × 10^−2^]
*k*_*D*_	—	0.017	[0, 1]	[0.016, 0.028]
*k*_*s*_	—	0.2	[0, 1]	[0.196, 0.211]

^1^ The confidence level is 95%.

^2^ *k*_1_ is the on-rate constant of monomer for the chemical reaction (1) in the cytoplasm. The other on-rate constants belong to the chemical reactions (5) underneath the plasma membrane.

^3^ It should not be set to zero because it is not quite small relative to other parameter values.

### Mutation of the N-terminal glycine residue of MA of Gag to alanine (Gag-G2A)

MA comprises the N-terminus of the Gag polyprotein, and it is responsible for targeting the Gag polyprotein to the PM. Therefore, mutation of the N-terminal glycine residue of MA to alanine (G2A) can reduce the attachment of a myristoyl group to Gag and impede its recruitment to the PM [44, 45]. In our model, the speed of Gag-G2A transport is slower compared with that of Gag WT. In addition, we assumed that *k*_*D*_ and *k*_*s*_ in Eq (6) for Gag-G2A were equal to the corresponding values for WT Gag. Therefore, we adjusted only one parameter *s*_1_ to fit the experimental data [10]. The value of this parameter is 2.20 × 10^−11^ *μm*/*s*, and its 95% confidence interval is [0, 0.09]. Because *s*1 is close to zero, we concluded that Gag-G2A might fail to hijack the molecular motor.

**Fig 5:**
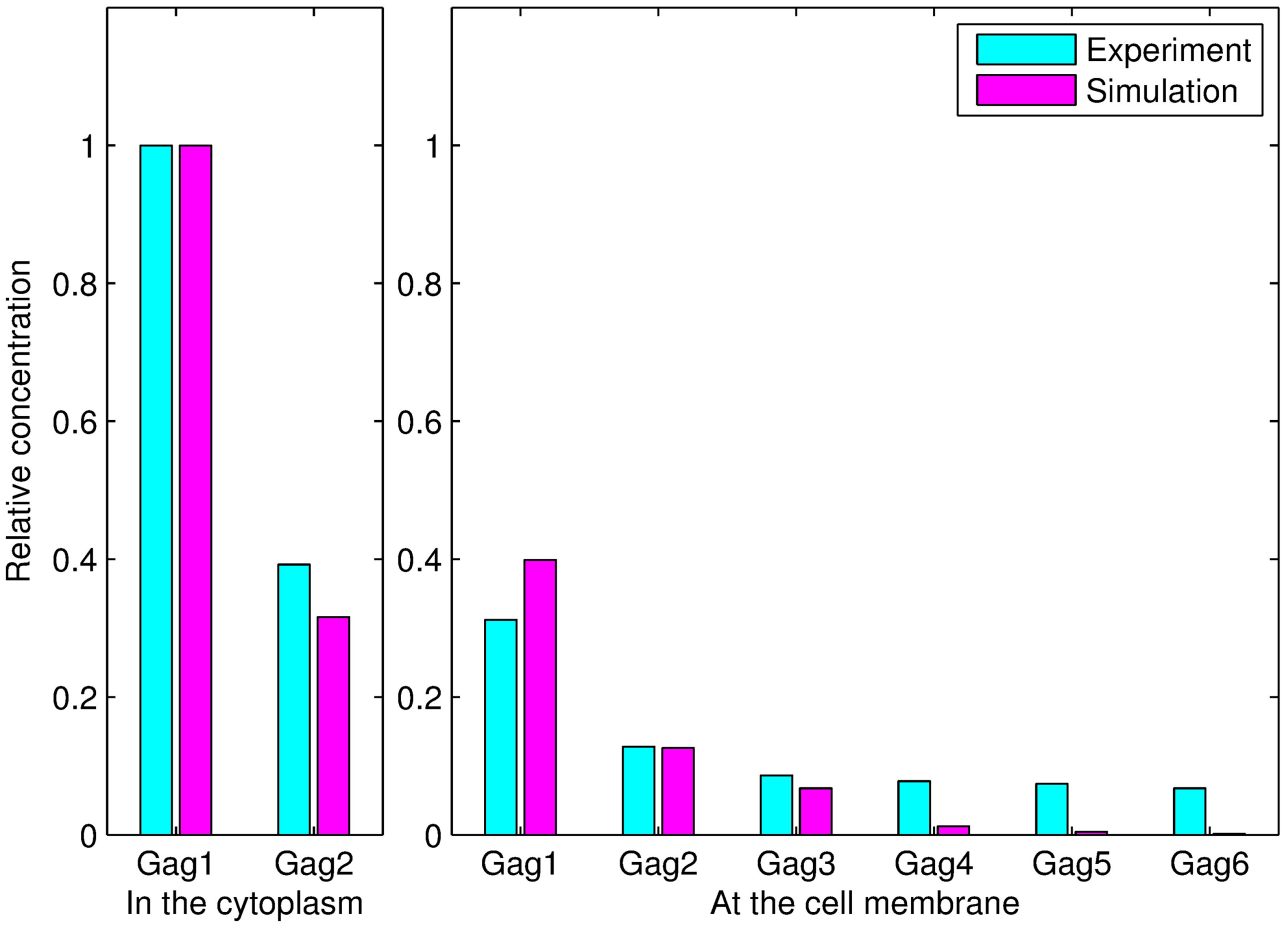
Comparison between the simulation results and experimental data for Gag-G2A. *Gagi*(*i* = 1, 2, ⋯, 6) denotes a polymer with *i* monomers. The left side illustrates the comparisons between the simulation and experimentally measured concentrations for monomers and dimers in the cytoplasm. The right side shows the differences between the simulation and experimentally measured concentrations with the six types of polymers at the plasma membrane. The concentration of each type of polymer was normalized by dividing by the concentration of Gag monomer in the cytoplasm.

The comparison between the simulation values and the experimental data, which is shown in Fig 5, clearly demonstrates that the simulation and experimental results are similar.

### Analysis of the identifiability of the parameters

In our study, we used eight data points for WT Gag and eight data points for Gag-G2A [10] to fit 13 parameters, including 10 polymerization coefficients *k*_*i*,*j*_, two proportionality coefficients *k*_*D*_ and *k*_*s*_ and the transfer speed *s*1 of Gag-G2A. The constraints for the 13 parameters are as follows:

- All 10 polymerization coefficients are positive.
- The two proportionality coefficients are between 0 and 1.
- The transfer speed of Gag-G2A is less than 1 *μm*/*s*.

After the parameter values are optimized based on experimental data [10], we evaluated how well the model parameters were determined by these data. In 2009, Raue et al. [46] proposed an approach to analyze the structural and practical identifiability of dynamical models by exploiting the profile likelihood, and this method has subsequently been widely applied in many fields, particularly the computational systems biology [47–50].

In this work, we used this technology [46] to analyze the identifiability of the parameters. First, finite sample confidence intervals for the parameters were estimated, and these are listed in Table 4. As shown, the confidence intervals of all of the parameters are within the bounds.

**Fig 6:**
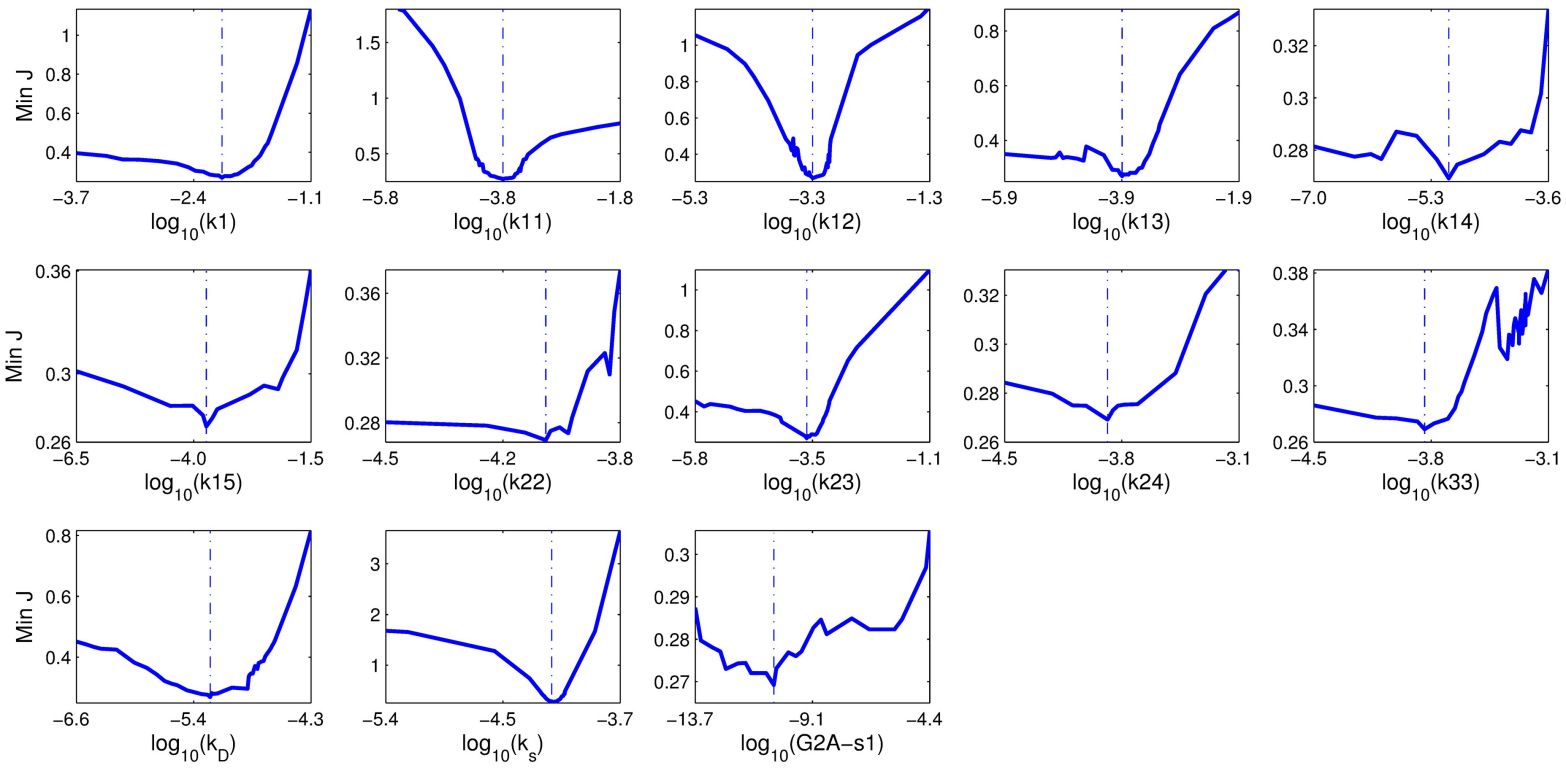
Blue lines display the profile likelihood for the parameter. The vertical dashed lines indicate the optimal values of the parameters shown in Table 4. Each parameter was varied over a wide range around its optimal value, and the remaining parameters were then refitted. All parameter values were log-transformed.

The profile likelihoods of all the parameters are shown in Fig 6. Specifically, the profile likelihoods for *k*11, *k*12, *k*23, *k*_*D*_ and *k*_*s*_ show a steep concave shape, indicating that the optimization route can rapidly reach the minimum. The profile likelihoods for *k*1, *k*15 and *k*24 also show a concave shape, however, the curves on the right side of the vertical dashed lines decrease slowly, indicating that their optimization routes might reach the minimum slowly. The profile likelihoods for *k*13, *k*14, *k*22, *k*33 and *G*2*A* − *s*1 have several local minima, hence, more iterations might be needed for the optimization route to jump out of and not get stuck at these local minima.

### “Knockout” mutations of the CTD of the CA of Gag (Gag-dCTD)

**Fig 7:**
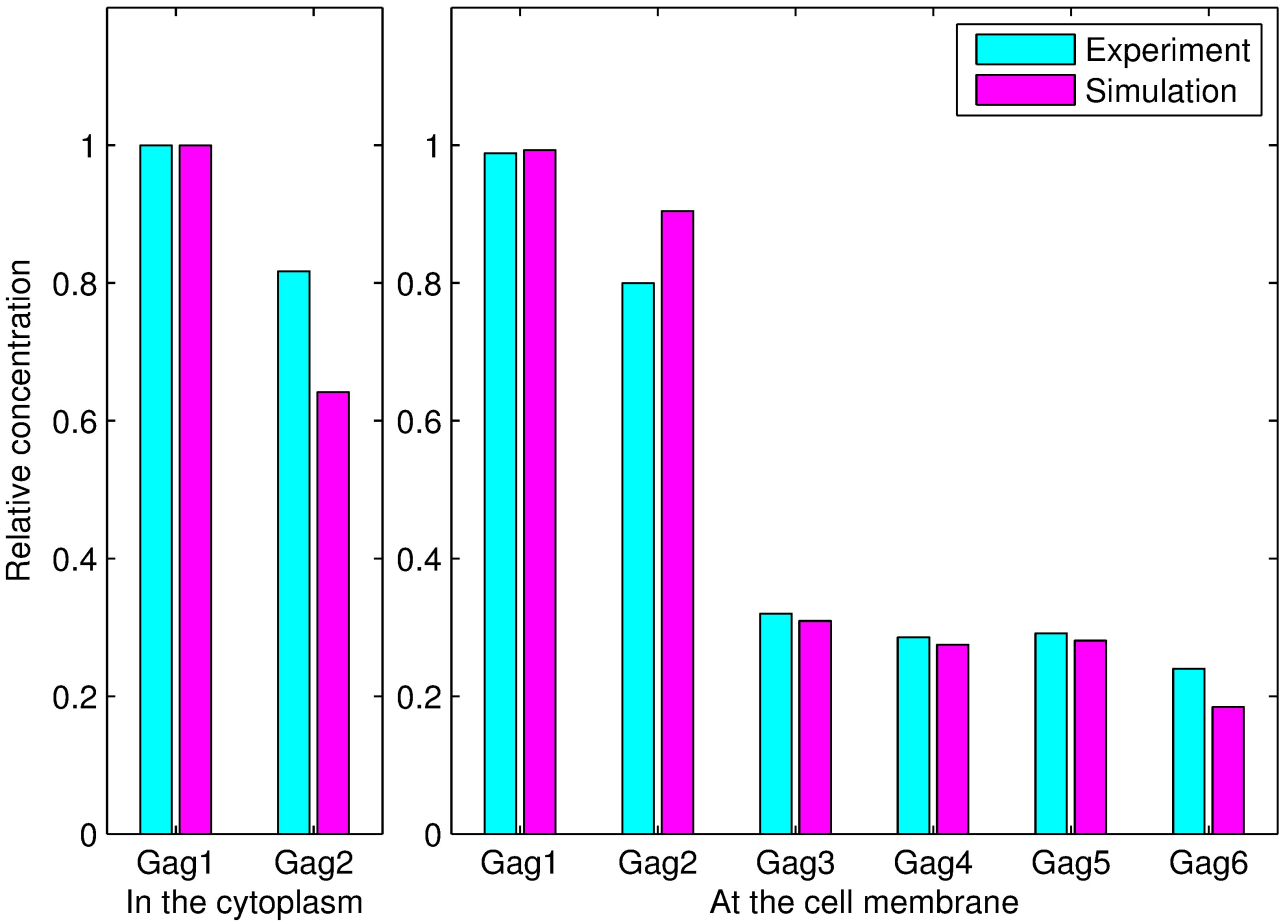
Comparison between the simulation results and experimental data for Gag-dCTD. *Gagi*(*i* = 1, 2, ⋯, 6) denotes a polymer with *i* monomers. The subfigure on the left side illustrates the comparisons between the simulation and experiment concentrations for monomers and dimers in the cytoplasm. The subfigure on the right side shows the differences between the simulation and experiment concentrations for the six types of polymers at the plasma membrane. The concentration of each polymer was normalized by dividing by the concentration of Gag monomer in the cytoplasm.

The CA is one of the four major domains of Gag and plays an important role in Gag multimerization and assembly at the PM. Furthermore, Gag: RNA binding is mediated by the CTD of the CA, which participates in Gag-Gag interactions. Thus, Gag-dCTD will show decreased on-rate constants. Furthermore, because the CA and MA of Gag are bound to each other, Gag-dCTD might show slightly impaired CA function. The damaged CA domain will slightly decrease the transport velocity of Gag, thus, the transport coefficient of Gag-dCTD might be slightly slower than that for WT Gag. This assumption is also supported by experimental data [10] for Gag-dCTD. Taken together, these assumptions indicate that the parameters *s*_1_, *s*_2_, *k*_*i*,*j*_ are decreased compared with the corresponding values for WT Gag. These limiting conditions were included in the process of parameter optimization. The values of these parameters are listed in Table 5, and the comparison between the simulation values and experimental data is shown in Fig 7. As shown, the numerical results agree with the experimental data.

**Table 5:** Values of some parameters for Gag-dCTD.

Parameter	Unit	Value	Parameter	Unit	Value
*k*_1_	*μm*^3^/(*ymol* ⋅ *s*)	7.22 × 10^−3^	*k*_15_	*μm*^3^/(*ymol* ⋅ *s*)	1.63 × 10^−4^
*k*_11_	*μm*^3^/(*ymol* ⋅ *s*)	9.13 × 10^−5^	*k*_22_	*μm*^3^/(*ymol* ⋅ *s*)	7.06 × 10^−5^
*k*_12_	*μm*^3^/(*ymol* ⋅ *s*)	1.53 × 10^−4^	*k*_23_	*μm*^3^/(*ymol* ⋅ *s*)	3.24 × 10^−4^
*k*_13_	*μm*^3^/(*ymol* ⋅ *s*)	5.15 × 10^−5^	*k*_24_	*μm*^3^/(*ymol* ⋅ *s*)	1.11 × 10^−4^
*k*_14_	*μm*^3^/(*ymol* ⋅ *s*)	8.12 × 10^−6^	*k*_33_	*μm*^3^/(*ymol* ⋅ *s*)	1.21 × 10^−4^
*s*_1_	*μm*/*s*	0.63			

### Budding and release time of VLPs

According to various references [21, 22, 51, 52], a VLP buds and releases after ∼6 hours.

In 2004, Briggs et al. [3] reported that the diameter of a VLP was ∼145 nm, and Carlson et al. [53] then found that a VLP was released with ∼2400±700 Gag proteins.

Jouvenet et al. [22] observed cells over a period of 30-60 minutes starting 5-6 hours after transfection and found that 50-150 puncta per cell typically appeared during this period. The behavior of these puncta could result in their classification into two discrete classes: slowly appearing puncta and rapidly appearing/disappearing puncta. The slowly appearing puncta represented the majority of events (74%) observed at 5-6 hours after transfection, and were indistinguishable from areas of the PM. The rapidly appearing/disappearing puncta were indistinguishable from endosomes. Therefore, Jouvenet et al. believed that the slowly appearing puncta might represent genuine VLPs assembly events.

Nermut et al. showed some pictures of VLPs in the budding state [21], and these images showed that most Gag proteins gathered to VLPs. Thus, the total Gag protein in the budding state at the PM can be estimated by the number of VLPs and Gag proteins per VLP. Taken together, these findings indicate that the threshold surface density of Gag at the PM can be computed as follows:

$$\begin{array}{c} \frac{\frac{NG}{6.023\times 10^{23}}\times 10^{24}\times Np\times p}{4\pi \times R^{2}}\;\left(unit:ymol/um^{2}\right) \end{array}$$ (14)

where *NG* is the number of Gag proteins in a VLP, *Np* is the total puncta per cell in the budding state, *p* is the ratio of slowly appearing puncta, and *R* is the radius of a cell.

When *NG* = 2400, *Np* = 50, *p* = 74% and *R* = 10, the threshold surface density is 1.17 × 10^2^ *ymol*/*um*^2^. When *Np* is changed to 100 and 150, the threshold surface densities are 2.34 × 10^2^ *ymol*/*um*^2^ and 3.51 × 10^2^ *ymol*/*um*^2^, respectively.

In our work, the surface density of Gag at the PM can be obtained by the following formula:

$$\begin{array}{c} S_{Gag}=\left(\sum_{i=1}^{6}i\times C_{Gagi}\right)*H\;\left(unit:ymol/um^{2}\right) \end{array}$$

where *S*_*Gag*_ denotes the surface concentration of Gag, *C*_*Gagi*_ denotes the volume concentration of *Gagi* underneath the PM, and *H* is the thickness of the concentrated domain of Gag underneath the PM, which is set to 0.13 *um* [5].

When the surface density of Gag is equal to the threshold surface density, the cumulative time is estimated as the budding and release time. Times of 3.51, 8.53 and 21.34 hours are predicted for threshold surface densities 1.17 × 10^2^, 2.34 × 10^2^ and 3.51 × 10^2^ *ymol*/*um*^2^, respectively. The predicted budding and release times agree with earlier findings to some degree [21, 51, 52], indicating that the total concentration of Gag underneath the PM is reasonable.

### Sensitivity and elasticity analysis

In 2011, Tavener et al. [54] defined the sensitivity of the *ith* model output variable *O*_*i*_(*P*, *T*) with respect to the *jth* parameter *P*_*j*_ at the time *T*, *S*_*i*,*j*_(*T*) as

$$\begin{array}{c} S_{i,j}\left(T\right)=\frac{\partial O_{i}\left(P,T\right)}{\partial P_{j}} \end{array}$$

These researchers also defined the elasticity of the *ith* output variable *O*_*i*_(*P*, *T*) with respect to the parameter *P*_*j*_, *E*_*i*,*j*_(*T*) as

$$\begin{array}{c} E_{i,j}\left(T\right)=\frac{P_{j}}{O_{i}\left(P,T\right)}\frac{\partial O_{i}\left(P,T\right)}{\partial P_{j}} \end{array}$$

Elasticity is defined in terms of relative sensitivity and can describe the rate of change in the relative size of the output variables with respect to the relative size of the parameters. An elasticity analysis would thus yield more reliable results. This definition has an extraordinarily wide range of applications [8].

**Fig 8:**
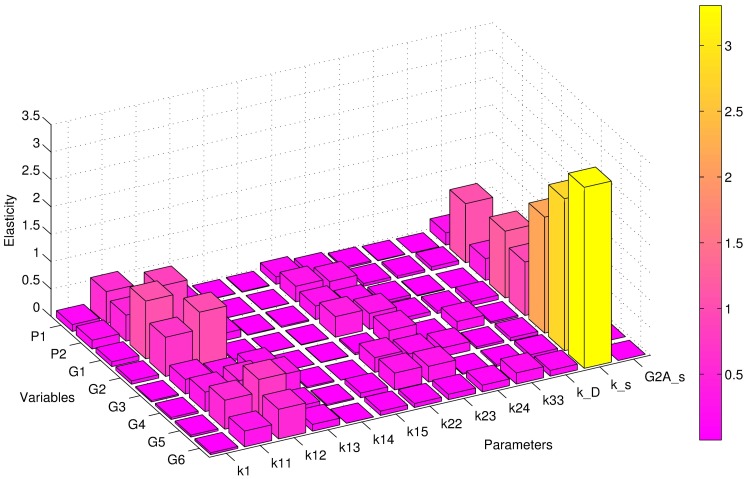
Elasticity analysis of parameters corresponding to eight types of polymers. *Gi*(*i* = 1, 2, ⋯, 6) denotes a polymer with *i* monomers at the plasma membrane. *P*1 and *P*2 indicate the monomer and dimer concentrations in the cytoplasm, respectively.

Based on the literatures [55, 56], the elasticity function *E*_*i*,*j*_(*T*) was estimated as follows:

$$\begin{array}{c} E_{i,j}\left(T\right)=\frac{P_{j}}{O_{i}\left(P,T\right)}\frac{\partial O_{i}\left(P,T\right)}{\partial P_{j}}\approx \frac{|O_{i}\left(P_{j}+\Delta P_{j},T\right)-O_{i}\left(P_{j}-\Delta P_{j},T\right)|/O_{i}\left(P_{j},T\right)\;}{(2\Delta P_{j}/P_{j}\;)\;} \end{array}$$

where Δ*P*_*j*_ is a small perturbation of the parameter *P*_*j*_. The elasticity values for all parameters corresponding to eight outputs, specifically the monomer and dimer concentrations in the cytoplasm and the concentrations of each of the polymers at the PM, are shown in Fig 8.

As shown in Fig 8, the elasticity values for the polymerization coefficients *k*11 and *k*12 are greater than those for the other polymerization coefficients. This findings indicates that perturbations of these two parameters can lead to relatively large changes in Gag polymer concentrations. Therefore, we can conclude that the corresponding two reactions, which involve the polymerization of two monomers to form a dimer and the polymerization of a monomer and a dimer to form a trimer, might be key reactions. If future drugs can decrease the values of *k*11 and *k*12, concentrations of Gag high-order polymers will be significantly reduced.

Among all of parameters, the highest elasticity value is found for *k*_*s*_, which measures the ability of Gag to land on the PM by active transport. Therefore, we can conclude that this process has a very significant impact on Gag polymers on the PM. As a result, suppression of the Gag concentration on the PM by reducing the ability of Gag to stay on the PM might be a good strategy.

The elasticity value of *k*_*D*_ is found to be very small, which illustrates that the landing of Gag on the PM by diffusion has little impact on changes in the concentrations of Gag polymers on the PM. In addition, the elasticity value for the transport speed *s*1 of Gag-G2A is very small with a value close to zero. This finding further supports the hypothesis that Gag-G2A might hardly be able to hijack molecular motors to move to the PM, and as a result, Gag-G2A might be an important drug target.

**Fig 9:**
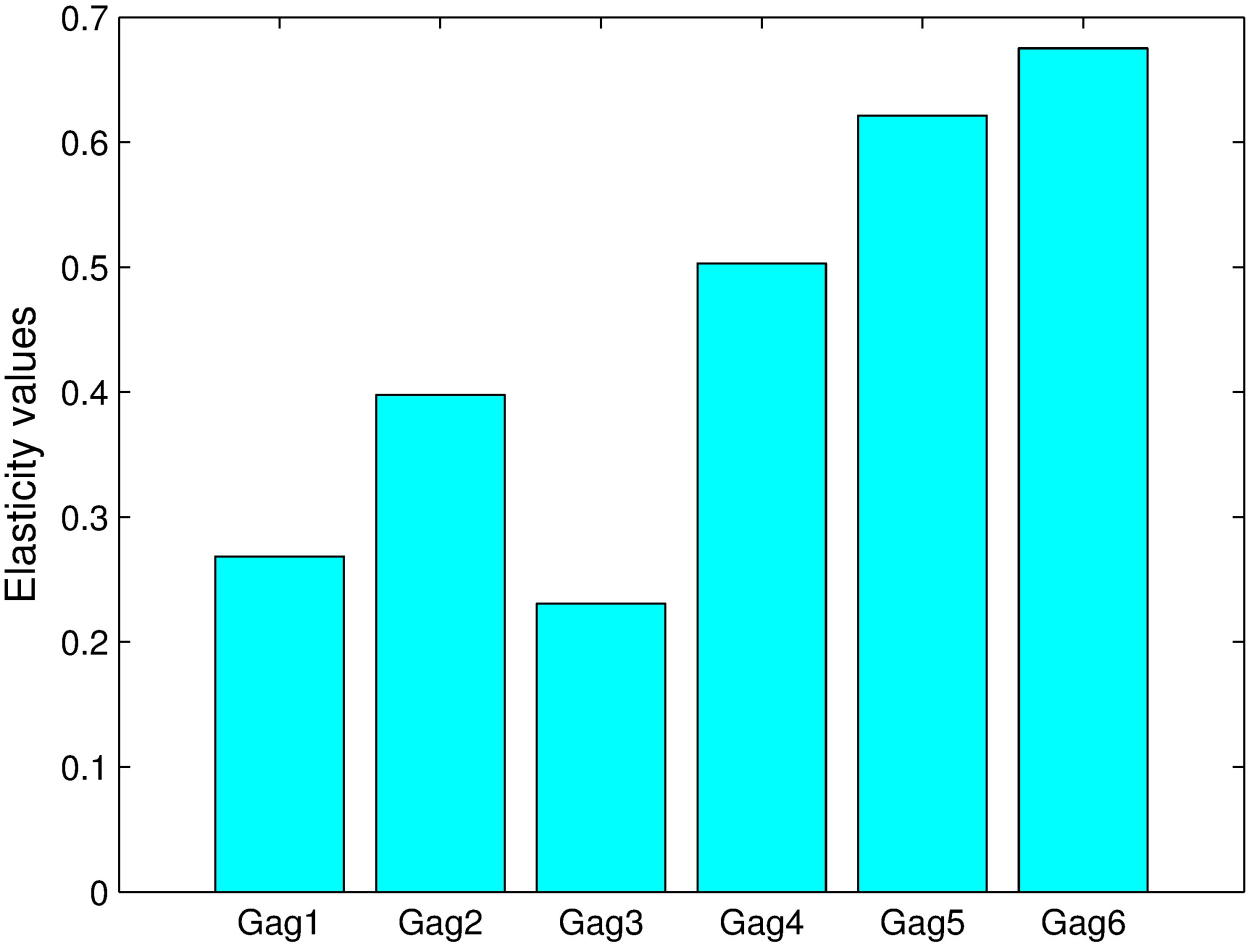
The global elasticity analysis of the parameters.

The global elasticity function was defined as follows:

$$GSA=\frac{1}{N}\overset{N}{\underset{k=1}{\sum }}\frac{\frac{\left|O\left(P+\Delta P^{k},T\right)-O\left(P,T\right)\right|}{O\left(P,T\right)}}{{\left\|\frac{\Delta P^{k}}{P}\right\|}_{\infty }}$$

where N is the total number of perturbations, and Δ*P*^*k*^ is the simultaneous perturbations of all parameters during the k-th perturbation. The global elasticity function values for 10% perturbations of all parameters are shown in Fig 9. As shown in the figure, perturbations of all the parameters are not sensitive to the output, which indicates that the proposed model is reasonable and robust.

### Analyzing the predominant pathways and the key intermediates in hexamer formation

The patterns underlying the formation of polymers constitute a very interesting topic [57, 58]. Using our model, we analyzed the weights of the pathways for the tetramer, pentamer, and hexamer formation.

Hexamer formation consists of three pathways:

$$\left(1\right)\;2Gag_{3}\overset{k_{33}}{\underset{k_{33}^{\prime }}{⇌}}Gag_{6}$$

$$\left(2\right)\;Gag_{2}+Gag_{4}\overset{k_{24}}{\underset{k_{24}^{\prime }}{⇌}}Gag_{6}$$

$$\left(3\right)\;Gag_{1}+Gag_{5}\overset{k_{15}}{\underset{k_{15}^{\prime }}{⇌}}Gag_{6}$$

The rate equation for the hexamer concentration can be described as follows:

$$\begin{array}{c} \frac{dP_{6}}{dt}=k_{33}P_{3}^{2}-k_{33}^{\prime }P_{6}+k_{24}P_{2}P_{4}-k_{24}^{\prime }P_{6}+k_{15}P_{1}P_{5}-k_{15}^{\prime }P_{6}-d_{6}P_{6} \end{array}$$

**Fig 10:**
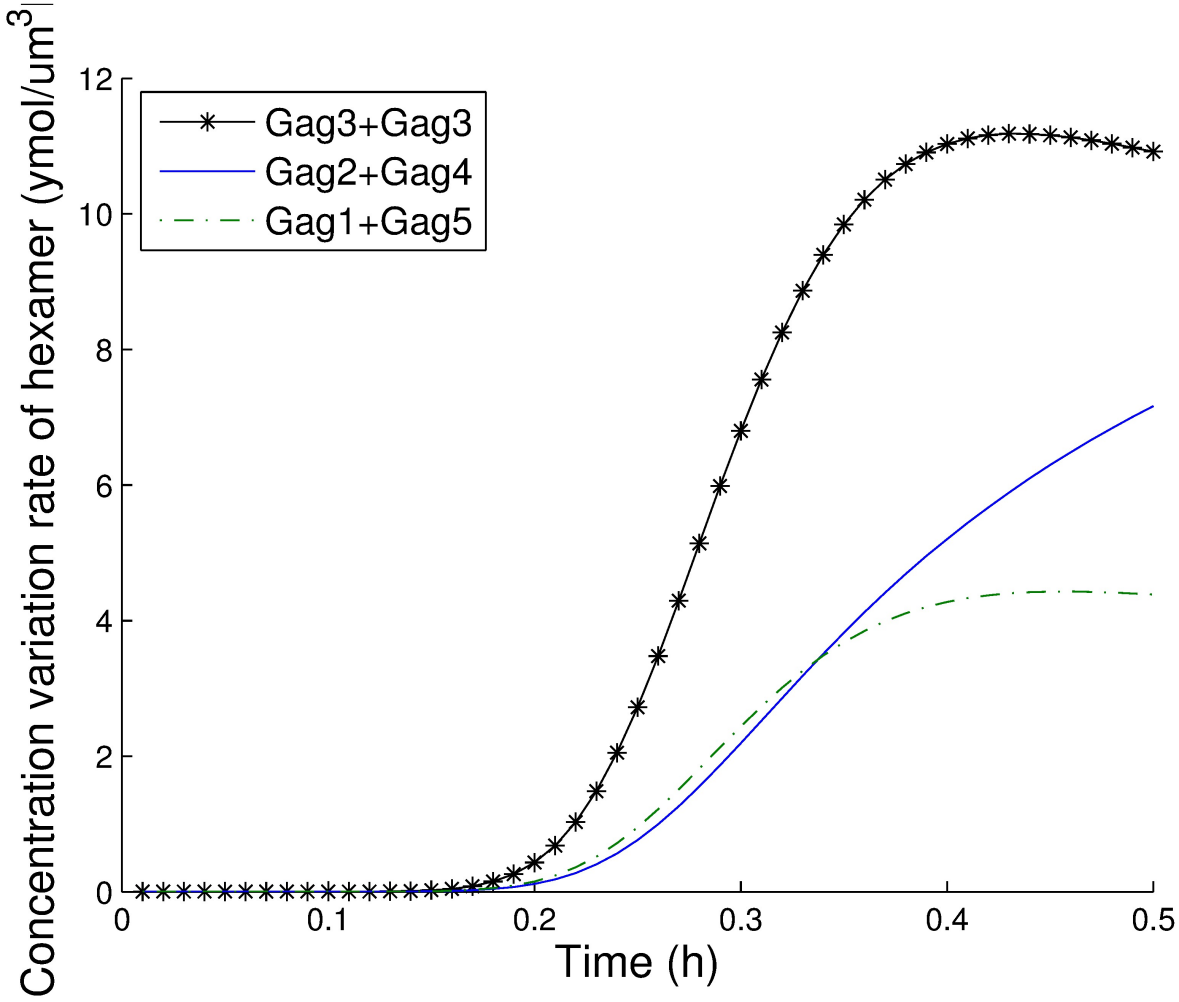
The contributions of three pathways to form hexamers.

**Fig 11:**
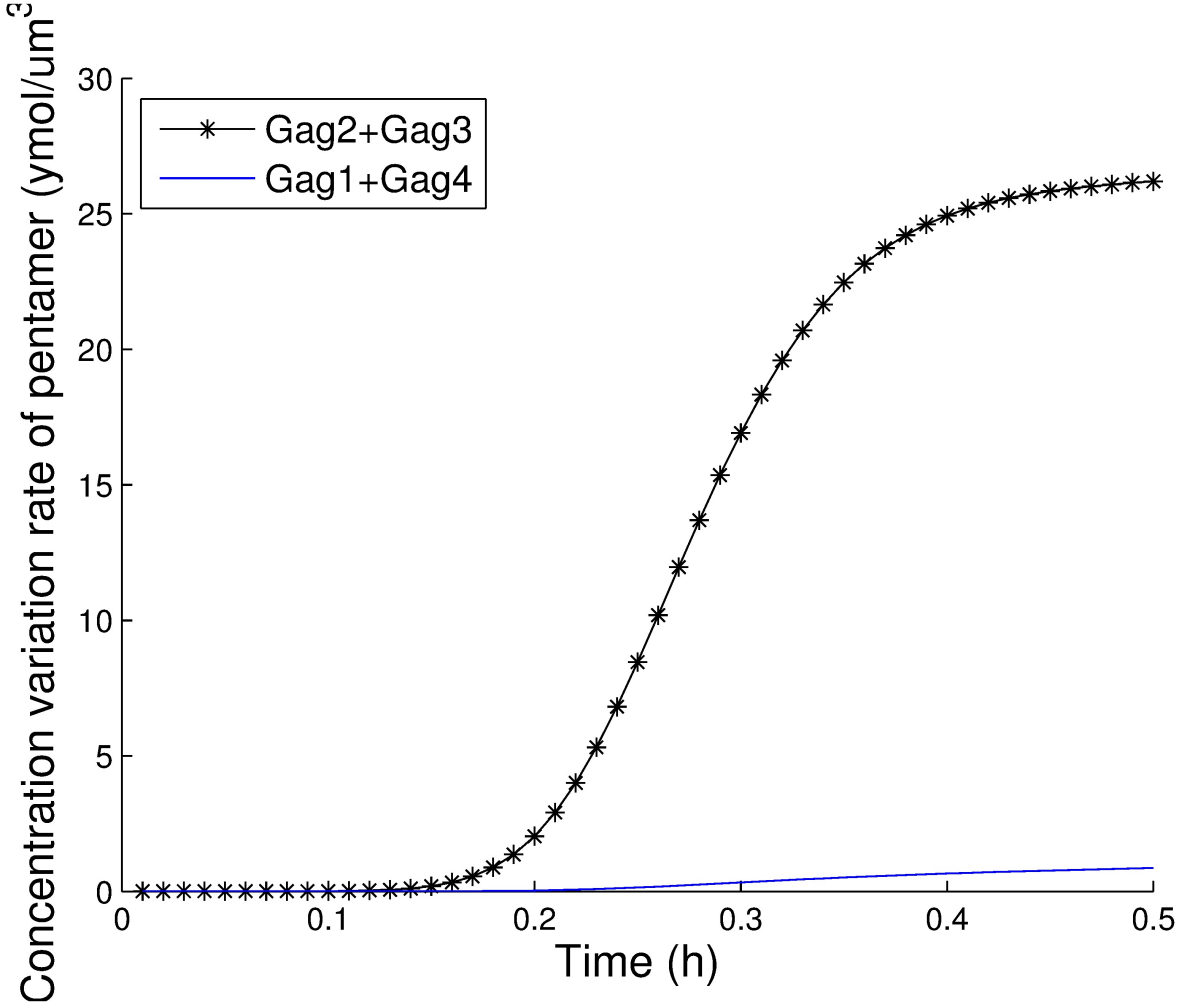
The contributions of two pathways to form pentamers.

**Fig 12:**
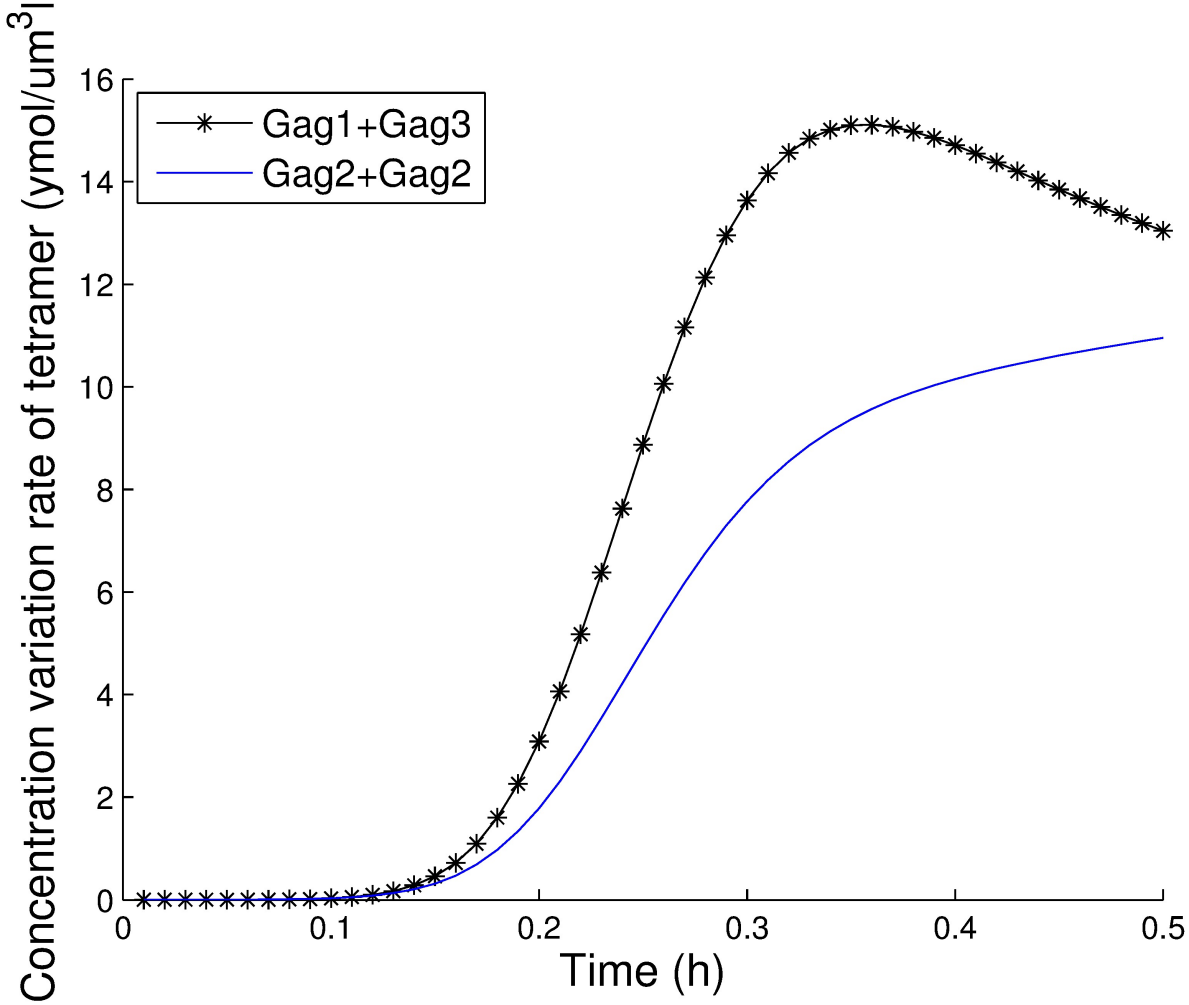
The contributions of two pathways to form tetramers.

The three pathways increase the hexamer concentration based on the following rates: $k_{33}P_{3}^{2}-{k^{\prime }}_{33}P_{6}$, *k*_24_ *P*_2_*P*_4_ − *k*′_24_*P*_6_ and *k*_15_*P*_1_ *P*_5_ − *k*′_15_*P*_6_. The largest value among these rates corresponds to the predominant pathway. The values for these three pathways during the first 30 minutes are shown in Fig 10, and the results clearly show that the first pathway is the most important after 12 minutes. Therefore, the predominant pathway is 2*Gag*_3_ ⇌ *Gag*_6_. We also explored the predominant pathways in pentamer and tetramer formation. As illustrated in Fig 11, the predominant pathway in pentamer formation is *Gag*_2_ + *Gag*_3_ ⇌ *Gag*_5_, and as shown in Fig 12, the predominant pathway in tetramer formation is *Gag* + *Gag*_3_ ⇌ *Gag*_4_. We compared these three most important pathways and found that the trimer intermediate was needed in all three predominant pathways. Therefore, we conclude that the Gag trimer might be a key intermediate in hexamer formation.

Kutluay et al. [10] revealed that a Gag trimer could not be formed in the cytoplasm and that a Gag trimer on the PM could not return to the cytoplasm. Therefore, the formation of a trimer indicates that the Gag protein complex can now stay on the PM. As shown in Figs 4, 5 and 7, the relative concentrations of trimers, tetramers, pentamers and hexamers are similar to each other. Therefore, we could infer that the concentrations of these high-order polymers depend heavily on the tirmer concentration. In addition, we reduced the trimer concentration to the corresponding value for Gag-G2A by increasing its degradation. The concentrations of the various Gag polymers are listed in Table 6, and as shown, tetramer, pentamer and hexamer concentrations decrease markedly. However, the same finding was not obtained for reductions in the tetramer and pentamer concentrations. Therefore, we can conclude that trimer formation, namely *Gag*_1_ + *Gag*_2_ ⇌ *Gag*_3_ might be a key pathway. As shown in Fig 10, the predominant pathway in direct hexmer formation is 2*Gag*_3_ ⇌ *Gag*_6_. Taken together, the key pathways for the formation of a hexamer from a monomer might be 2*Gag*_1_ ⇌ *Gag*_2_, *Gag*_1_ + *Gag*_2_ ⇌ *Gag*_3_ and 2*Gag*_3_ ⇌ *Gag*_6_, and the key intermediates in hexamer foramtion might be the dimer and trimer polymers.

**Table 6:** Compare the concentrations of higher-order polymers ^1^.

	P1	P2	G1	G2	G3	G4	G5	G6
WT Gag	6.95	3.31	6.85	6.21	5.42	4.95	4.84	4.31
Gag-G2A	5.54	1.75	2.21	0.70	**0.37**	**0.07**	**0.03**	**0.01**
Reduce G3 concentration	6.68	4.18	6.37	8.20	**0.37**	2.71	0.58	0.88
Reduce G4 concentration	6.61	3.63	6.25	7.00	5.32	**0.07**	4.74	3.10
Reduce G5 concentration	7.50	3.21	7.83	5.85	5.49	4.92	**0.03**	2.63
Reduce G6 concentration	6.70	3.23	6.41	6.07	5.07	4.35	3.55	**0.01**

^1^ P1 and P2 indicate the monomer and dimer concentrations in the cytoplasm, respectively. Gi (i = 1, 2, ⋯, 6) denotes a polymer with *i* monomers on the PM. The unit is *μm*^3^/*ymol*.

### Four theoretical combined methods for suppressing the Gag concentration

In the wake of developments in basic science, many of the most promising HIV drugs in clinical development do not target specific retroviral enzymes but rather act by interrupting the assembly of viral factors with host proteins [59]. For example, some agents that disrupt protein-protein interactions during the entry of HIV-1 are showing great clinical potential [59]. However, according to AIDSinfo, no clinically available drug can inhibit Gag transport and assembly (https://aidsinfo.nih.gov/drugs/Search/a-z/all), and the development of these agents is a daunting challenge. Because the current treatments for HIV-1 normally include the use of multiple drugs in an attempt to control this virus, we proposed and analyzed four theoretical combined methods for inhibiting Gag transport and assembly based on our model. These analyses might be helpful to the design of new anti-AIDS drug.

We took three approaches, specifically the degradation rates for Gag polymers, Gag-G2A and Gag-dCTD, into account and designed the following four theoretical combined methods:

C1: Increasing all degradation rates of the polymers by 50% and setting *s*1 and *s*2 to the means of the corresponding values for WT Gag and Gag-G2A.
C2: Increasing all degradation rates of the polymers by 50% and setting *s*1, *s*2 and *k*_*i*,*j*_ to the means of the corresponding values for WT Gag and Gag-dCTD;
C3: Setting *s*1 and *s*2 to the means of the corresponding values for WT Gag and Gag-G2A and setting *k*_*i*,*j*_ to the mean of the corresponding values for WT Gag and Gag-dCTD.
C4: Increasing all degradation rates of polymers by 50%, setting *s*1 and *s*2 to the means of the corresponding values for WT Gag and Gag-G2A, and setting *k*_*i*,*j*_ to the mean of the corresponding values for WT Gag and Gag-dCTD.

**Fig 13:**
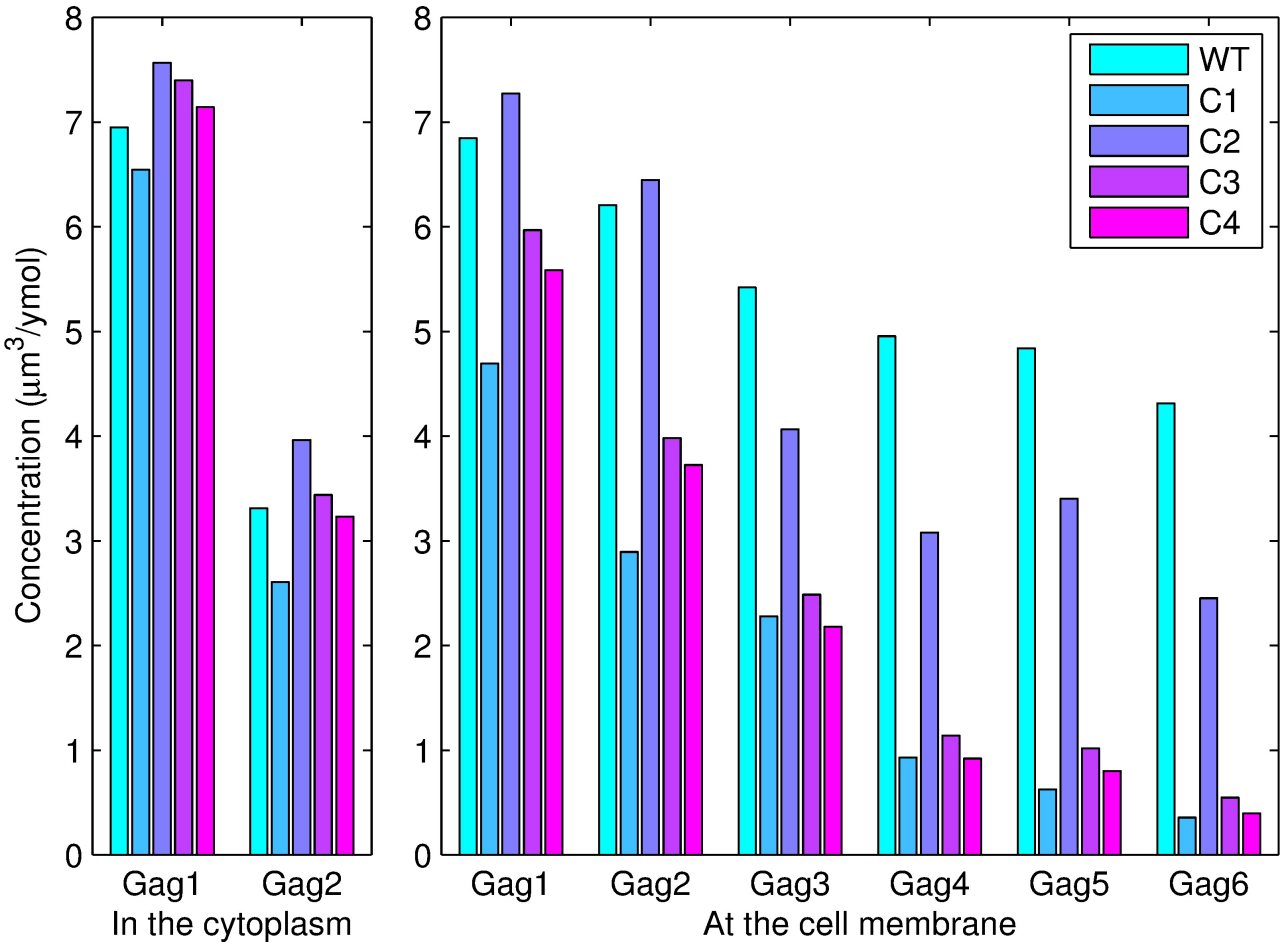
Concentration of polymers in the cytoplasm and PM during the first 30 minutes of simulations using the four theoretical combined methods and WT Gag. *Gagi*(*i* = 1, 2, ⋯, 6) denotes a polymer with *i* monomers.

For these four theoretical combined methods, we computed the concentrations of Gag polymers in the cytoplasm and PM during the first 30 minutes, and the corresponding results are shown in Fig 13. As shown in the figures, the concentrations of high-order polymers (e.g., tetramer, pentamer and hexamer) are relatively lower with methods C1, C3 and C4. We also found that these three methods involved the Gag-G2A mutation. Therefore, we speculate that the N-terminal glycine residue of the MA of Gag might be a promising drug target.

### Reducing the concentrations of higher-order polymers of Gag-G2A and WT Gag

Gag-G2A significantly decreases the concentrations of Gag higher-order polymers and thus might be a potential key drug target. To explore the mechanisms responsible for decreasing the concentrations of higher-order polymers of Gag-G2A, we first reduced the trimer concentration of WT Gag to 0.37 *μm*^3^/*ymol* by increasing the trimer degradation rate to yield the the corresponding concentration of Gag-G2A trimers. The concentrations of Gag polymers are shown in the fourth line in Table 6. Simelarly, we decreased the tetramer, pentamer and hexamer concentrations to the corresponding low concentrations for Gag-G2A, respectively, and the results are listed in Table 6.

After reducing the trimer concentration in Table 6, we compared the tetramer, pentamer and hexamer concentrations with those of WT Gag. The concentrations of these higher-order polymers were all markedly decreased, particularly the hexamer concentration. As shown by the results, the tetramer, pentamer and hexamer concentrations are all very dependent on the trimer formation. However, the same does not hold true for the tetramer and pentamer polymers. Therefore, these results further support the conclusion that the Gag trimer might be a key intermediate and that trimer formation might be a key pathway.

In addition, we compared the tetramer, pentamer and hexamer concentrations obtained with Gag-G2A after reducing the trimer concentration listed in the fourth line in Table 6. The resulting concentrations were markedly higher than those found for Gag-G2A. Thus, Gag-G2A does not use decrease the polymerization coefficients to decrease the polymer concentrations.

Compared with Gag-G2A, the monomer and dimer concentrations for WT Gag in the cytoplasm are reduced by approximately a quarter and a half, respectively, and the monomer, dimer, tetramer, pentamer and hexamer concentrations for WT Gag on the PM are reduced by approximately 68%, 88%, 93%, 98%, 99% and 99%, respectively. On the PM, Gag-G2A reduces the monomer and dimer concentrations by reducing their active transport speeds, which results in decreases in the concentrations of higher-order polymers on the PM. In the cytoplasm, the low transport speeds of monomers and dimers for Gag-G2A and their very weak diffusions lead to the high monomer and dimer concentrations, and most of these monomers and dimers are gathered near the perinuclear area. However, due to the high degradation rates, these high concentrations near the perinuclear area show rapid reductions. Therefore, in the entire cytoplasm, these monomer and dimer concentrations are ultimately lower than those found for WT Gag.

## Discussion

In this study, we developed a model to simulate the intracellular trafficking and polymerization of HIV-1 Gag protein. The model parameters were fitted using published experimental data [10]. The profile likelihoods of these parameters were used to show their identifiability, and an elasticity analysis of these parameters was used to show the robustness of this model. The model was able to predict the budding and release time of a VLP, and the results were in agreement with the findings of some previous studies [21, 51, 52]. Moreover, the model could also be applied to two mutated versions: Gag-dCTD and Gag-G2A. Using our model, we analyzed the weight of the pathways involved in the polymerization reactions, and concluded that the Gag dimer and trimer might be two key intermediates in hexamer formation. Moreover, we inferred that the three key pathways in the formation of a hexamer from a monomer might be the polymerization of two monomers to form a dimer, the polymerization of a monomer and a dimer to form a trimer, the polymerzation of two trimers to form a hexamer. We also explored four theoretical combined methods for suppressing the Gag concentration and concluded that the N-terminal glycine residue of the MA of Gag might be a proming drug target.

There is no denying that the presented modeling approach is merely an approximation to reality. However, it successfully provides a consistent and quantitative description of the transport and polymerization of Gag and lays a broad foundation for further developments. Future experimental and theoretical research is required to support the various assumptions employed in the model.

A number of important questions have not been fully addressed and need for further examination. For instance, there are two types of motor proteins: one conveys cargo to the nucleus, and the other conveys cargo to the PM. We consider only the average velocity of the transport of cargo to simplify the model. Thus, it is important to address the transport processes of these two types of motor proteins in future studies. In addition, a dynamical analysis [60] of HIV-1 trafficking process and a multilayer networks analysis [61] related to HIV-1 will also be investigated in future work.
